# Microglial infiltration mediates cognitive dysfunction in rat models of hypothalamic obesity *via* a hypothalamic-hippocampal circuit involving the lateral hypothalamic area

**DOI:** 10.3389/fncel.2022.971100

**Published:** 2022-08-22

**Authors:** Chong Song, Wei Wei, Tong Wang, Min Zhou, Yunshi Li, Bing Xiao, Dongyi Huang, Junwei Gu, Linyong Shi, Junjie Peng, Dianshi Jin

**Affiliations:** ^1^Department of Neurosurgery, The Central Hospital of Dalian University of Technology, Dalian, China; ^2^Department of Neurosurgery, Nanfang Hospital, Southern Medical University, Guangzhou, China; ^3^Department of Neurosurgery, The Third Hospital of Mianyang (Sichuan Mental Health Center), Mianyang, China; ^4^The First School of Clinical Medicine, Southern Medical University, Guangzhou, China

**Keywords:** hypothalamic obesity, memory dysfunction, lateral hypothalamic area, microglial cells, neural circuits

## Abstract

This study aimed to explore the mechanism underlying cognitive dysfunction mediated by the lateral hypothalamic area (LHA) in a hypothalamic-hippocampal circuit in rats with lesion-induced hypothalamic obesity (HO). The HO model was established by electrically lesioning the hypothalamic nuclei. The open field (OP) test, Morris water maze (MWM), novel object recognition (NOR), and novel object location memory (NLM) tests were used to evaluate changes in cognition due to alterations in the hypothalamic-hippocampal circuit. Western blotting, immunohistochemical staining, and cholera toxin subunit B conjugated with Alexa Fluor 488 (CTB488) reverse tracer technology were used to determine synaptophysin (SYN), postsynaptic density protein 95 (PSD95), ionized calcium binding adaptor molecule 1 (Iba1), neuronal nuclear protein (NeuN), and Caspase3 expression levels and the hypothalamic-hippocampal circuit. In HO rats, severe obesity was associated with cognitive dysfunction after the lesion of the hypothalamus. Furthermore, neuronal apoptosis and activated microglia in the downstream of the lesion area (the LHA) induced microglial infiltration into the intact hippocampus *via* the LHA-hippocampal circuit, and the synapses engulfment in the hippocampus may be the underlying mechanism by which the remodeled microglial mediates memory impairments in HO rats. The HO rats exhibited microglial infiltration and synapse loss into the hippocampus from the lesioned LHA *via* the hypothalamic-hippocampal circuit. The underlying mechanisms of memory function may be related to the circuit.

## Introduction

Hypothalamic obesity (HO) is a common perioperative complication of sellar region disease. Trauma, tumors, surgery and other factors that damage the hypothalamic nuclei can lead to disorders of energy regulation mechanisms and persistent hyperthyroidism (Poretti et al., 2004; Müller et al., 2022); thus, HO patients frequently experience simultaneous abnormalities in blood glucose, blood pressure and other indicators, which seriously affect their prognosis (Heinks et al., 2018). Patients with hypothalamic lesions, especially those with HO, are more likely to exhibit memory dysfunction than non-obese patients (Poretti et al., 2004). In previous studies, patients with hypothalamic lesions were found to have severely impaired cognition and memory; alterations in 12 of the 20 variables assessing cognition and memory were statistically significant (Fjalldal et al., 2013). However, a strong link has yet to be established between memory mechanisms and hypothalamic projections.

Our previous work elucidated the roles of microglial cells in the hypothalamus after injury to the pituitary stalk and evaluated metabolic function in patients with hypothalamic damage (Feng et al., 2018a; Zhou M. F. et al., 2019; Ou et al., 2020). Extending this line of research, in the present study, we investigated how lesion-induced HO may contribute to cognitive decline *via* the hypothalamic-hippocampal neural circuit and microglial infiltration.

In this study, we first investigated the role of the arcuate nucleus (ARC) and the ventromedial nucleus of the hypothalamus (VMH) in feeding behavior and cognitive function by lesioning these nuclei. The lateral hypothalamic area (LHA) receives projections from the ARC and VMH (Elias et al., 1999; Valassi et al., 2008; Hahn and Swanson, 2015), and is generally regarded as a critical regulator of feeding, motivated behavior and arousal. The LHA is a large heterogeneous structure and many neural circuits transmit signals through the LHA including the hypothalamic-hippocampal circuit (Kelley et al., 2019). Despite accumulating evidence supporting the role of the hypothalamus in memory regulation (Hsu et al., 2015), how information is represented *via* the hypothalamic-hippocampal circuit impacts memory remains unclear (Jimenez et al., 2018).

Emerging research suggests an intriguing interaction between neural circuitry and the immune system (Cilz et al., 2019; Zhou W. et al., 2019), wherein dysregulated communication could disrupt neuroimmune homeostasis and induce neuropathology (Rapuano et al., 2020; Kodali et al., 2021; Madeira et al., 2022; So et al., 2022). For example, an increase in microglial can indicate a neural response to circulating proinflammatory signals and may mediate neuroinflammatory responses (De Luca et al., 2016), which have been linked to synapse loss and cognitive decline in both human and animal studies (Opitz, 2014; Linehan et al., 2020; Shi et al., 2021; Wu et al., 2021). However, to date, no study has examined the impact of lesion-induced HO on microglial polarization within the brain region that regulates cognitive function.

Therefore, in the current study, we established a mature HO model with memory dysfunction induced by hypothalamic injury, and identified the neural circuits and microglial infiltration that mediate memory impairment.

## Materials and methods

### Animals

Male Sprague–Dawley (SD) rats (weighing 200–230 g, average body weight 220 g) were obtained from the Animal Center of Southern Medical University. The animals were housed separately in plastic boxes (48 cm × 35 cm × 25 cm) under standard conditions (12 h light/dark cycle, at 22–26°C, and with a humidity of 40–60%) and provided with a standard chow diet and clean water *ad libitum*. After the lesion surgery, rats were given free access to food and water at an ambient temperature of 22 ± 1°C with a 12 h light/dark cycle and housed individually. Body weight and food intake were recorded regularly. All the experiments were performed in compliance with ARRIVE (Animal Research: Reporting *in vivo* Experiments) Guidelines2.0 and strictly followed the 3 Rs (Replacement, Reduction, and Refinement) to avoid unnecessary sacrifice. This experiment was approved by the Ethics Committee of Nanfang Hospital.

### Lesion surgery

For the lesion surgery, a stereotaxic apparatus was used. The coordinates of the ARC and VMH were determined based on previous research by our research group and the Paxinos and Watson rat brain atlas (Roth et al., 2011; Feng et al., 2018a; Zhou M. F. et al., 2019; Zhou et al., 2021). The Bregma point (AP: –2.6 mm, ML: ±0.6 mm, DV: –9.6 mm) was used as the target lesion point. A dental drill and a lesion needle were used to penetrate the skull; the needle was pressed vertically against the top of the skull to the extent possible. The ARC and VMH were lesioned bilaterally by passing a current of 1.5 mA through the anode for 25 s. In the sham-operated group, the needle was inserted from the same position on the top of the skull as in the HO group to a depth of 9.6 mm; no electricity was applied. All animals were housed in independent cages after the surgery, and body weight and food intake were regularly recorded every day. The recording method was as follows: the remaining food from each cage was weighed at a regular time every day and added to 80 g, and the daily food intake was calculated as (80-remaining rat food) in grams. The linear distance between the tip of the rat’s nose and the anus was recorded on the 28th day as the body length of the rat, and an increase in body adiposity was indicated by an increase in the Lee index, a marker of adiposity calculated by dividing the cube root of body weight (g) × 10 by naso-anal length (mm).

### Hematoxylin and eosin staining

The rats were deeply anesthetized by intraperitoneal injection of 1% sodium pentobarbital solution, perfused with normal saline until the liver turned white, and then fixed with 4% paraformaldehyde (POM) by constant injection until the rat’s body turned rigid. POM was fixed for 24–48 h. Paraffin sections were prepared and stained with Hematoxylin and eosin (H&E) by hematoxylin staining for 3 min, rinsing with double distilled water, differentiating with 1% hydrochloric acid alcohol solution for 3 s, rinsed with double distilled water, and then staining with eosin for 1 min. The slides were placed in an oven at 60°C to dry, sealed with gum, and photographed using an upright fluorescence microscope (Olympus, BX63). According to the HE staining results, the rats with Off-target lesions were excluded.

### Open field test

The open field test (OF) imitates unsafe surroundings, evaluates animals’ autonomous behavior, and reveals how tense the animals are (Cheng et al., 2022; Hou et al., 2022; Wang L. P. et al., 2022; Wen et al., 2022). A 30 min dark adaptation was conducted before initiating the test. Then, one rat at a time was carefully placed in the center of an open field box (35 cm × 45 cm × 35 cm) to record the total traveled distance and the route for 10 min. The field was sanitized with 75% ethanol solution before the initiation of the following test.

### Morris water maze test

The spatial learning and memory of rats were evaluated with the Morris water maze (MWM) (Deng et al., 2019; Liang et al., 2022; Wang L. P. et al., 2022). Training took place for the first 5 day; testing took place on the 6th day. During training, the rat was placed in the MWM pool facing the pool wall. Each training trial lasted 60 s. After finding the platform, the rat was allowed to stay on it for 30 s. If the rat did not find the platform, they were guided to the platform and allowed to stay there for 30 s. Each rat was allowed to rest for 10 min between training sessions. The pool was divided into 4 equal quadrants; the edge of the midpoint of each quadrant was used as a water entry point, for a total of 4 water entry points. For each rat, the escape latency, distance traveled, swim path, etc., were recorded each time they found the platform during training. At testing, the rats were placed in the pool (lacking a hidden platform) at a water entry point and allowed to swim freely for 60 s. During this time, the rat’s latency to first reach the original platform area, its swimming duration and distance traveled in each quadrant, the number of times it passed the original platform area, and its swim path were recorded.

### Novel object recognition and novel object location memory tests

Both tests were conducted in the same box as described in section “Morris water maze test.” All animals were habituated to the testing arena for 5 min for 2 consecutive days before the familiarization phase of the test (Bagheri et al., 2022; Florido et al., 2022). For familiarization: in the novel object recognition (NOR) test, one rat was placed into the arena with two identical sample objects (A, B) and allowed to explore for 5 min; in the novel object location memory (NLM) test, the rat was placed in the center of the same box that had two identical sample objects (A, B) and allowed to explore for 5 min. The NOR test was performed after a 24-h period. Object B was replaced by a new object C. Object C was put in the same spot as object B. They were given 5 min to freely explore the box. The NLM test was performed after a 24-h period. Object A remained in the same position as before and object B was relocated to a different location in the open field box. They were allowed 5 min to freely explore the box.

The time spent by the rats investigating each object or position during the test session was used to measure the NOR and NLM. “a” and “b” reflect the time spent discovering the familiar and the new object/location, respectively. We calculated the variables e = a + b and d = (b-a)/e. The “e” variable reflects the total duration exploring all objects and locations. The “d” variable is a discrimination index that differentiates between new and familiar objects/locations, as well as a relative measure of discrimination that takes exploratory activity into account (e). Exploration was defined as sniffing approximately a distance of 2 cm or touching the objects with the nose and/or forepaws. Exploratory activity was not described as sitting on or turning around the objects. The animals that explored the items for less than 5 s were not included in the study.

### Neural circuit tracing

The rats were anesthetized and placed into the stereotaxic apparatus. To trace the neural circuit, the skull was exposed through a small incision (Lu et al., 2022), according to the target brain area coordinates for the LHA (bregma: AP = –3.2 mm, LV = ±1.6 mm, DV = –8.6 mm). A small hole was drilled at this site, a glass injection pole was inserted into the brain tissue, and CTB488 was injected with an air pressure system (Fasanella et al., 2008; Liu X. et al., 2022). A micromanipulator was used to control the injection speed, fixing it at 25 nl/min, and the pipette was removed 5 min after the injection of the required amount of CTB488. After injection of CTB488, the brains were harvested after perfusion in the dark for 2 weeks. The brain slices were cut and photographed using an upright fluorescence microscope (Olympus, BX63), and the images were analyzed with ImageJ software. According to the HE staining results, the rats with Off-target CTB488 injection sites were excluded.

### Immunofluorescence

As above, the rats were deeply anesthetized by intraperitoneal injection of 1% sodium pentobarbital solution, perfused with normal saline until the liver turned white, and then fixed with 4% paraformaldehyde by constant instillation. POM was fixed for 24–48 h. The LHA and hippocampal layers were removed and dehydrated with 15 and 30% sucrose solutions, respectively. Dehydration was complete when the brain tissue settled to the bottom of the container. The brain tissue was then embedded in optimal cutting temperature (OCT) gel, and after being rapidly frozen at –80°C, brain slices were cut with a cryostat (Leica, CM1850UV) with a thickness of 20–30 μm (Wang X. et al., 2020). Rat brain slices at the LHA and hippocampal levels were selected, rinsed with phosphate-buffered saline (PBS), blocked with 5% goat serum solution at room temperature for 2 h, immersed in the corresponding primary antibody solutions (Iba1, SYN, PSD95, NeuN, and Caspase3), and incubated overnight at 4°C. On the second day, after rinsing in the dark with 5% PBS with Tween (PBST), the cells were soaked in the corresponding fluorescent secondary antibody conjugated with Alexa-488 (Thermo Fisher Scientific, Waltham, MA, United States), incubated in the dark at room temperature for 2 h, and rinsed in the dark with 5% PBS. Then, the slice was flattened, and DAPI-conjugated anti-fluorescence quenching mounting agent (Abcam, ab104139) was applied. The slides were imaged and photographed using an upright fluorescence microscope (Olympus, BX63), and the images were statistically analyzed with ImageJ software.

### Image analysis and quantification

Fluorescent images were analyzed using ImageJ software. For analysis of the microglial number, Iba1^+^, NeuN, and Caspase cells were measured manually in the immunostaining channel using ImageJ. For quantification of the microglial soma size, a polygonal region of interest containing only one soma was selected, and microglial soma size was measured manually in the immunostaining channel using ImageJ (Ramírez et al., 2020). For the co-localization of Iba1 with SYN and PSD95 analysis, after background subtraction to remove camera noise, contrast enhancement was performed to increase the signal intensity and remove low-level background fluorescence, and the microglial soma contained the SYN/PSD95 was measured using the ImageJ (Nelson et al., 2007; Lutz et al., 2021).

### Western blotting

Rats were perfused with ice-cold normal saline, and the hippocampal tissue was collected under a stereomicroscope, washed thoroughly with PBS, lysed with radio-immunoprecipitation assay (RIPA) buffer (50 mM Tris-HCl, pH 8.0; 1 mM EDTA, pH 8.0; 5 mM DTT; and 2% SDS) containing protease inhibitor and phosphoric-acid protease inhibitor using a low temperature tissue homogenizer, and kept at 4°C for 30 min (Feng et al., 2018b). The protein concentration was determined by a bicinchoninic acid (BCA) assay (Beyotime Inc., Xiamen, China). Protein samples were subsequently separated using SDS–PAGE and electrotransferred to polyvinylidene difluoride membranes (Millipore, Burlington, MA, United States). After blocking with 5% bovine serum albumin (BSA), the membranes were incubated with primary antibodies overnight at 4°C and then incubated with the corresponding horseradish peroxidase-conjugated secondary antibody for 1 h at room temperature. Finally, the signal was detected using enhanced chemiluminescence reagents (Millipore, United States) and captured with a digital camera, and the images were statistically analyzed with ImageJ software.

### Antibodies

We used the following antibodies: NeuN (Abcam, ab279296), Caspase3 (Proteintech, 19677-1-AP), Iba1 (Abcam, ab153696), GAPDH (10494-1-AP, Proteintech Group), PSD95 (Novus, NBP2-12872), SYN (Novus, NBP2-61895), CTB488 (Thermo Fisher Scientific, C34775), and DAPI (Abcam, ab104139).

### Statistics

SPSS 26.0 was used for data analysis. The presence of significant differences between each group (sham-operated group and HO group) in this study was determined using a two-tailed Student’s *t*-test for the experiments. Data are presented as the mean ± SD or mean ± SEM. The statistical significance was set as *P* < 0.05.

## Results

### Obesity in rats after arcuate nucleus/ventromedial nucleus of the hypothalamus lesioning

**FIGURE 1:**
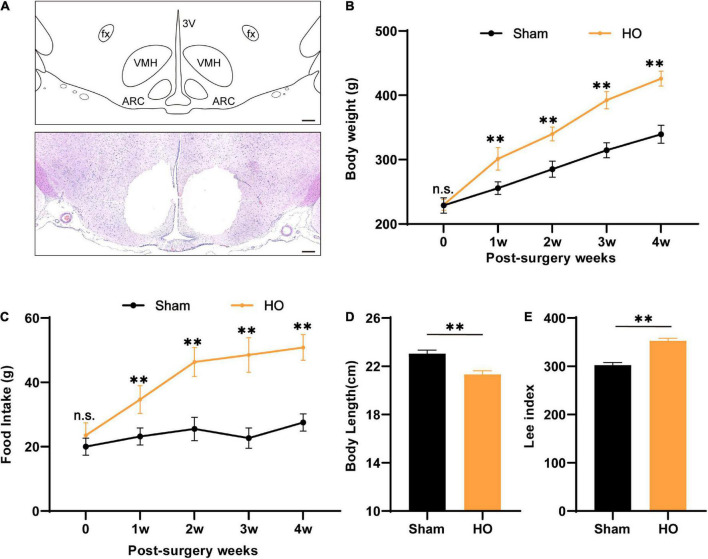
Characteristics of biological parameters after ARC and VMH lesion surgery. **(A)** Schematic overview of the mediobasal hypothalamus and coronal brain sections (–2.6 mm from the bregma) from electrolytic lesions in the ARC and VMH. Scale bars, 200 μm. **(B,C)** Dynamic changes in postoperative body weight and food intake of rats in the sham-operated group and HO group during the experiment, *n* = 6, **P < 0.01 vs. sham-operated group. **(D,E)** Body length and Lee’s index of rats in each group at 28 day after operation, *n* = 6, **P < 0.01 vs. sham-operated group. All data are presented as the M ± SD of each group. ARC, arcuate nucleus; VMH, ventromedial nucleus of the hypothalamus; 3V, 3 ventricle; fx: fornix column.

According to previous clinical studies, the ARC and VMH are often damaged by the compression of hypothalamic lesions or intraoperative injury (Bougnères et al., 2008; Roth et al., 2011). Therefore, we used electrical injury to the bilaterally ARC and VMH to construct the HO rat model (Figure 1A). After the model-construction operation, we recorded changes in the body weight and food intake of rats for 28 day. Compared with the sham-operated group, the body weight and food intake of the HO group increased significantly. During the entire experimental period, the body weight and food intake of the HO group were significantly higher than those of the sham-operated group (Figures 1B,C). On the 28th day after the operation, we recorded the body weight, daily food intake, and body length of the rats and calculated the changes in the Lee index of the rats in each group. On the 28th day, the weight of the HO rats was higher than that of the sham-operated rats (Figure 1B, *P* < 0.01), and more than 20% of the rats in the sham-operated group met the criteria for obesity over this same period; the food intake of the HO rats (50.8 ± 4.0 g/day) was significantly higher than that of the sham-operated rats (Figure 1C, *P* < 0.01), but the body length of the HO rats was shorter than that of the sham-operated rats (Figure 1D, *P* < 0.01). In addition, the Lee index of the rats in the HO group was significantly higher than that of the rats in the sham-operated group (Figure 1E, *P* < 0.01), suggesting that rat models of HO can be constructed by this operation method.

### Hypothalamic injury leads to postoperative memory dysfunction in the Morris water maze test of hypothalamic obesity rats

**FIGURE 2:**
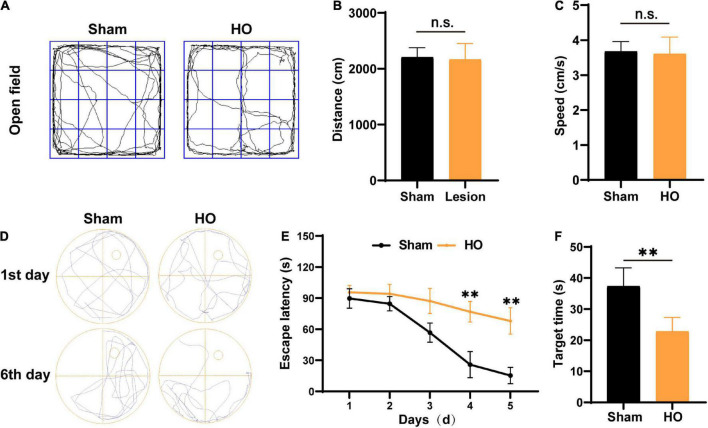
Verification of the HO rat model using the OP test and MWM test. **(A)** Movement path in the OP test. **(B,C)** The total moving distance and movement speed traveled by rats in each group. **(D)** Swim path in the MWM test. **(E)** The escape latency of rats in each group, *n* = 6, **P < 0.01 vs. sham-operated group. **(F)** The latency to reach the original location of the platform of rats in each group, *n* = 6, **P < 0.01 vs. sham-operated group. All data are presented as the M ± SEM of each group.

According to clinical studies, obesity is often accompanied by cognitive and memory dysfunction in humans (Bougnères et al., 2008; Fjalldal et al., 2013). Therefore, we used the OP and MWM to evaluate the cognitive and memory function of the HO rats. The traveling trace of the distance (cm) and speed (cm/s) of rats were detected in an open field for 10 min. No significant difference was found in the distance (Figures 2A,B) and speed (Figure 2C) of the sham-operated rats and the HO rats. Next, after 5 day of MWM training, the rats were tested to evaluate their spatial learning and memory. The swim paths of rats in the sham-operated group and the HO group are shown in Figure 2D. Compared with the sham-operated group, the escape latency of the HO group was prolonged during days 3–5 of the training period (Figure 2E, *P* < 0.01). During testing (day 6), the HO group spent a shorter amount of time in the target quadrant than the sham-operated group (*P* < 0.01, Figure 2F). These results indicate that the HO rats exhibited obvious cognitive and memory impairments.

### Hypothalamic injury leads to postoperative memory dysfunction in the novel object recognition and recognition location memory tests of hypothalamic obesity rats

**FIGURE 3:**
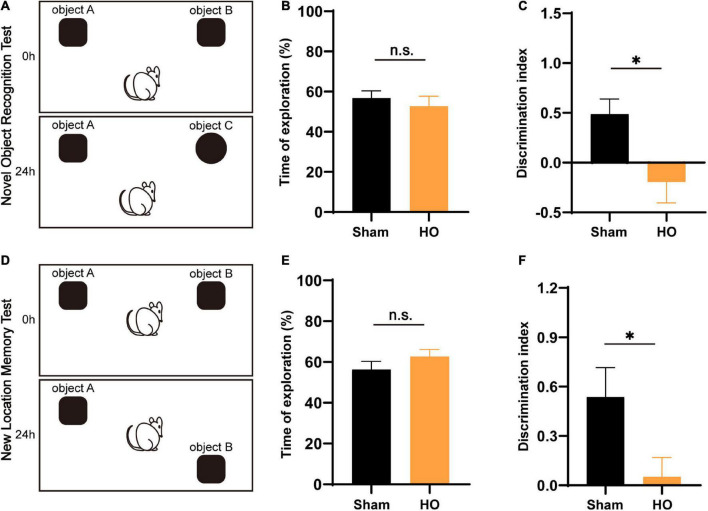
Effects of lesions on recognition memory assessed by the NOR test and NLM test. **(A)** Schematic diagram of the novel object recognition (NOR) test setup. **(B,C)** Total exploratory time (%) and recognition index (%) in each group, *n* = 6, *P < 0.05 vs. the sham-operated group. **(D)** Schematic of the novel location memory (NLM) test setup. **(E,F)** Total exploratory time (%) and recognition index (%) in each group, *n* = 6, *P < 0.05 vs. the sham-operated group. All data are presented as the M ± SEM of each group.

Novel object recognition and NLM tests are widely accepted to assess hippocampal-dependent learning and memory processes. The NOR and NLM results are illustrated in Figure 3. For total object exploration, significant effects were not observed between the two groups in either the NOR or NLM tests (Figures 3B,E). In the NOR test, HO rats had dramatically fewer contacts with the novel object compared to the sham-operated group (*P* < 0.05, Figure 3C). In the NLM test, the analysis of discrimination index data showed that HO animals displayed a lower discrimination index than the sham-operated group (*P* < 0.05, Figure 3F). These results indicate that the HO rats exhibited disordered hippocampal-mediated working memory.

### Inflammatory infiltration in the hippocampus *via* the hypothalamic-hippocampal circuit

**FIGURE 4:**
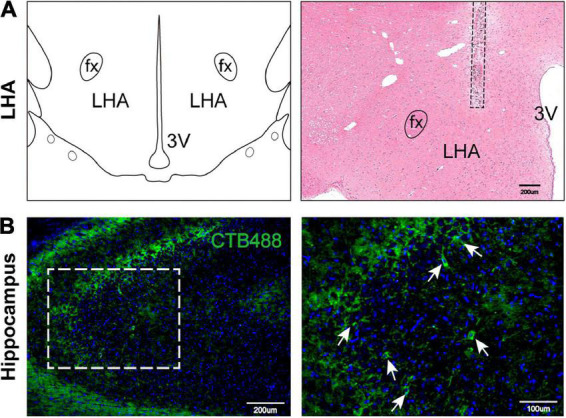
Hypothalamic-hippocampal neural circuit. **(A)** Schematic representation of the microinjection site in the LHA on a coronal plane. H&E staining indicates the CTB488 injection site. LHA, lateral hypothalamic area; fx, fornix column; 3V, third ventricle. **(B)** The expression of marked hippocampal cells projecting to the LHA. Marked cells (green) and DAPI (blue). Scale bars, 200 μm for low magnification images and 100 μm for high magnification images. LHA, lateral hypothalamic area; 3V, 3 ventricle; fx: fornix column.

To explore the cause of the cognitive and memory impairments in HO rats, we speculated that a hypothalamic-hippocampal neural circuit may link the LHA and the hippocampus. To verify the existence of this circuit (Fasanella et al., 2008), we injected CTB488 into the LHA for neural circuit tracing. We assessed whether the hippocampus contained cells expressing green fluorescence (due to the hypothalamic injection) and found obvious neurons in the hippocampus positive for this fluorescence (Figures 4A,B), which suggests the existence of a hypothalamic-hippocampal circuit.

**FIGURE 5:**
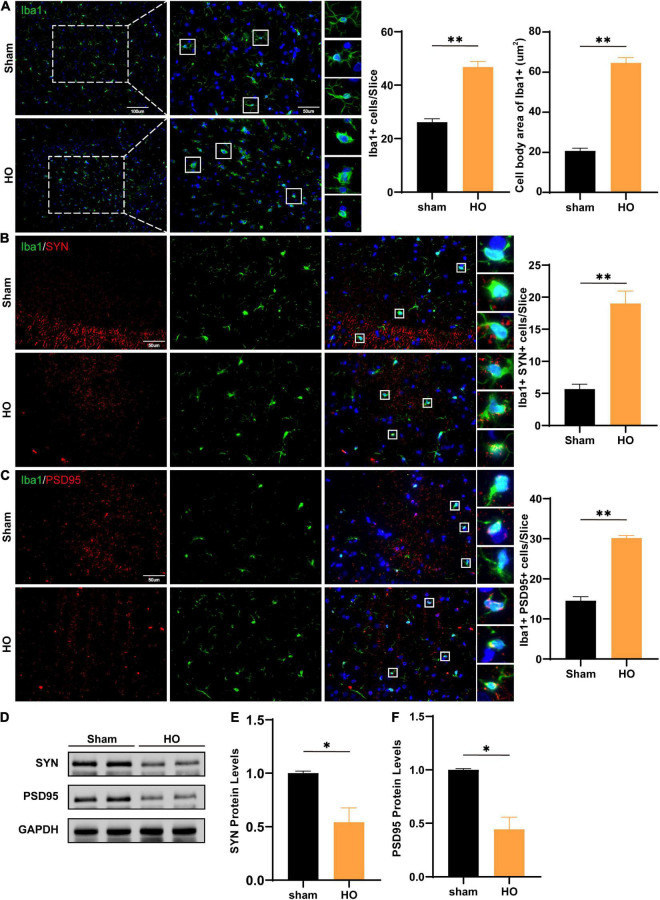
Microglial cells and SYN/PSD95 protein in the hippocampal region of HO rats. **(A)** Immunofluorescence staining of Iba1^+^ cells. Iba1 (green) and DAPI (blue). Scale bars, 100 μm for low magnification images and 50 μm for high magnification images; quantification of Iba1^+^ cells and the cell body area in the hippocampus, *n* = 4–6, **P < 0.01 vs. sham-operated group. **(B)** Immunofluorescence staining of Iba1^+^ and SYN in the hippocampus: SYN (red), Iba1 (green), and DAPI (blue); scale bars, 50 μm, *n* = 6, **P < 0.01 vs. sham-operated group. **(C)** Immunofluorescence staining of Iba1^+^ and PSD95 in the hippocampus: PSD95 (red), Iba1 (green), and DAPI (blue); scale bars, 50 μm, *n* = 6, **P < 0.01 vs. sham-operated group. **(D–F)** The protein levels of SYN and PSD95 in the hippocampus, *n* = 3, *P < 0.05 vs. sham-operated group. All data are presented as the M ± SEM of each group.

Next, we detected inflammatory cells infiltrating the hippocampus in the two groups and found that compared with those in the sham-operated group, there were an increase in the hippocampal glial cells and the soma of Iba1^+^ cells in the HO group (*P* < 0.01, Figure 5A). Synaptic plasticity in the hippocampus is required for cognition (Fang et al., 2022; Hösli et al., 2022); therefore, we evaluated the co-expression of the microglial and synaptic-related proteins, the presynaptic and postsynaptic markers synaptophysin (SYN) and postsynaptic density protein 95 (PSD95). The results showed increased SYN-immunoreactive and PSD95-immunoreactive materials inside the microglial, suggesting there was more engulfment of synaptic components in HO rats (*P* < 0.05, Figures 5B,C). Furthermore, Western blotting showed that SYN protein (*P* < 0.05, Figures 5D,E) and PSD95 protein (*P* < 0.05, Figures 5D,F) in the hippocampus were both reduced in the HO group. And the synapse engulfment may be the underlying mechanism by which the remodeled microglial mediates memory dysfunction in HO rats.

### Inflammatory infiltration in the lateral hypothalamic area in hypothalamic obesity rats accompanied by neuronal apoptosis

**FIGURE 6:**
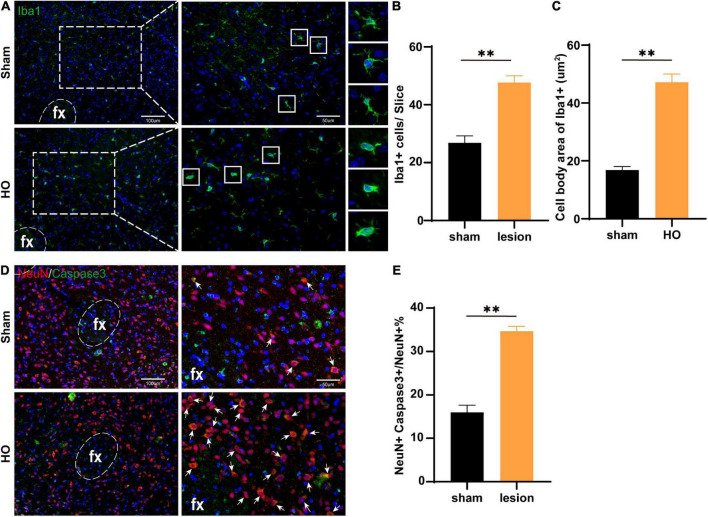
Microglial cells in the LHA region of obese rats were accompanied by neuronal apoptosis. **(A)** Immunofluorescence staining of Iba1^+^ cells. Iba1 (green) and DAPI (blue). Scale bars, 100 μm for low magnification images and 50 μm for high magnification images. **(B,C)** Quantification of Iba1^+^ cells and the cell body area in the LHA, *n* = 4–6, **P < 0.01 vs. sham-operated group. **(D)** Immunofluorescence in the LHA: NeuN (red), Caspase3 (green), and DAPI (blue); scale bars, 100 μm for low magnification images, 50 μm for high magnification images. **(E)** Quantification of neuronal apoptosis in the LHA, *n* = 6, **P < 0.01 vs. sham-operated group. All data are presented as the M ± SEM of each group.

Furthermore, we searched the LHA, which is downstream of the injured nuclei, to identify the mechanism underlying differences in spatial memory. We detected microglial by immunohistochemical staining and found that compared with the sham-operated group, the HO group exhibited a significant amount of microglial infiltration in the LHA, with increased soma of the Iba1^+^ cells (*P* < 0.01, Figures 6A–C). To determine whether inflammatory cells led to apoptosis in the LHA, we further examined neuronal apoptosis in the LHA; compared with that in the sham-operated group, the proportion of Caspase3^+^ neurons in the LHA of the HO group was significantly increased (*P* < 0.01, Figures 6D,E). Therefore, HO rats exhibited massive neuronal apoptosis in the LHA, an effect that may lead to memory dysfunction through the hypothalamic-hippocampal circuit with hippocampal microglial infiltration.

## Discussion

Obesity is caused by dysregulation of energy metabolism, a process that is finely controlled by the central nervous system (CNS); the hypothalamus, in particular, has emerged as the master regulator of whole-body energy homeostasis (Seoane-Collazo et al., 2015). Hormonal and nutrient-sensing hypothalamic nuclei coordinate central and peripheral responses to maintain normal body weight, food intake, energy expenditure, and nutrient distribution (Valassi et al., 2008; Suzuki et al., 2011; Müller, 2017). In these nuclei, specialized neuronal populations are interconnected to transmit and receive information from different extrahypothalamic brain regions, coordinating whole-body energy homeostasis (Lubaczeuski et al., 2015).

Hypothalamic injury-induced obesity is due to complex and unclear region-specific mechanisms involving nuclei, neurons, neuropeptides and neural circuits in multiple hypothalamic regions (Fouda et al., 2021); however, the ARC and VMH are the first areas affected and the primary areas of involvement according to previous clinical studies (Müller, 2017). Therefore, given previous animal models and studies (Roth et al., 2011), we constructed lesion-induced rat models by targeting the bilateral ARC and VMH to simulate HO without damaging the hippocampus. These HO rats showed a typical pattern of obesity, with significant increases in food intake and body weight, after surgery.

Hypothalamus damage often causes cognitive and memory impairments in patients (Poretti et al., 2004; Fjalldal et al., 2013). To mimic this clinical phenomenon, many studies have examined the connection between obesity and cognitive impairment, but these obesity models mainly utilized HFD and transgenic methods (Poon, 2020; Vargas-Rodríguez et al., 2022); few focused on the hypothalamic injury like the ARC/VMH-lesioned models. So there was little discussion on the potential contribution of the ARC and the VMH themselves to cognitive dysfunction, and the underlying mechanism is still unclear. Several studies have indicated that local inflammation in the hypothalamus caused by the HFD, altering internal hypothalamic circuitry and hypothalamic outputs to other brain regions. The result is a disruption to cognitive function mediated by regions such as the hippocampus, amygdala, and reward-processing centers (Miller and Spencer, 2014; Tan and Norhaizan, 2019).

The hippocampus is believed to regulate cognition and memory (Abu-Elfotuh et al., 2022; Chancey and Howard, 2022); damage to the neural structure of the hippocampus or changes in the local microenvironment may lead to cognitive and memory impairments (Aniol et al., 2022; Chen et al., 2022; Keinath et al., 2022).

Notably, in the present study, we demonstrated that hypothalamic damage is sufficient to cause impairments in cognition and memory, even with an intact hippocampal structure. Previous physiological studies have shown that the hypothalamus, especially the LHA, has intricate connections with other brain regions, including the midbrain, thalamus, diencephalon, brainstem, and hippocampus (Lee et al., 2021). The hypothalamic nuclei and the hippocampus are linked through neuronal projections and regulatory circuits. Therefore, we speculated that in lesion-induced HO, concurrent severe memory impairment would be related not only to pathological changes in hypothalamic nuclei but also to the *trans-*cerebral area linking the LHA and the hippocampus. Considering the complex neural projections within the hypothalamus, like the Papez circuit (Aggleton et al., 2016; Xia et al., 2020), the LHA-hippocampal circuit could be one of the explanations.

The complexity and plasticity of synaptic connections within the brain provide the neurophysiological basis of learning and memory (Rebola et al., 2017; Liu D. et al., 2022). Our results showed a high amount of microglial infiltration in the LHA and the hippocampus, accompanied by neuronal apoptosis in the LHA. Neuroinflammation may cause degeneration of the hypothalamus (Kim et al., 2017), and these pathological features may be linked to cognitive impairment *via* hypothalamic-hippocampal circuits. Increased immune responses could contribute to cognitive dysfunction, as demonstrated previously. Recent studies have shown that microglial are key regulators of synaptic pruning and play a key role in synaptic remodeling, plasticity, and laying out neural circuits (Xu et al., 2021; Wang M. et al., 2022). Microglial were found to engulf synapses during light-induced degeneration in a photic injury model (Xu et al., 2021). Synaptic connections in the brain are highly dynamic and variable in strength and connectivity (Xu et al., 2021). One study showed that microglial eliminate synaptic components in the adult hippocampus, whereas depleting microglial or inhibiting phagocytosis of microglial prevents forgetting, which suggests that synapse elimination by microglial leads to dissociation of engrams and the forgetting of previously learned contextual fear memory (Wang C. et al., 2020). Therefore, the “inappropriate repair” of synapses by microglial may affect the stability of synaptic connections in neural circuits, thereby affecting cognitive function. Synapse elimination by microglial may thus lead to degradation of memory engrams and forgetting of previously learned contextual fear memory.

In addition, the number of responding microglial cells depended on the severity of the injury. In general, only microglial cells in the immediate vicinity of the microlesion were activated, whereas cells farther away (> 90 μm) did not respond or did not immediately respond (Nimmerjahn et al., 2005). Therefore the infiltration we found in the hippocampus of HO rats may be the mechanism underlying the hypothalamic-hippocampal circuit. Furthermore, selective modulation of microglial activity in the LHA and/or hippocampus could be a potential approach to mimic or even modulate the cognitive effect of HO rats.

In summary, we explored the cognitive and memory impairments associated with lesion-induced HO in rats and found that microglial infiltrated the LHA and hippocampus; this infiltration may be the mechanism underlying the hypothalamic-hippocampal circuit. Our results showed that projection from the hippocampus to the LHA was involved in cognition in HO rats.

## Data availability statement

The original contributions presented in this study are included in the article/supplementary material, further inquiries can be directed to the corresponding authors.

## Ethics statement

This animal study was reviewed and approved by the Ethics Committee of Nanfang Hospital, Southern Medical University.

## Author contributions

CS, JP, and DJ contributed to the conception and design of the study and funding acquisition. CS, WW, TW, and MZ designed the figures, performed the experiments, and wrote the manuscript with supervision from CS, JP, and DJ. YL, BX, DH, JG, and LS did the investigation, and collected and analyzed the data. All authors contributed to the article and approved the submitted version.

## Abbreviations

PSD95: postsynaptic density protein 95
SYN: synaptophysin
Iba1: ionized calcium binding adaptor molecule 1
NeuN: neuronal nuclear protein
3V: 3 ventricle
LHA: lateral hypothalamic area
ARC: arcuate nucleus
VMH: ventromedial nucleus of the hypothalamus
HO: hypothalamic obesity
fx: fornix column
OP: open field
MWM: Morris water maze
NOR: novel object recognition
NLM: novel object location memory
CTB488: cholera toxin subunit B conjugated with Alexa Fluor 488
POM: paraformaldehyde.
